# Highly efficient *ex vivo* lentiviral transduction of primary human pancreatic exocrine cells

**DOI:** 10.1038/s41598-019-51763-z

**Published:** 2019-11-01

**Authors:** Jeetindra R. A. Balak, Natascha de Graaf, Arnaud Zaldumbide, Ton J. Rabelink, Rob C. Hoeben, Eelco J. P. de Koning, Françoise Carlotti

**Affiliations:** 1 Department of Internal Medicine, Leiden University Medical Center, Leiden, the Netherlands; 2 Department of Cell and Chemical Biology, Leiden University Medical Center, Leiden, the Netherlands; 3 Hubrecht Institute, Utrecht, the Netherlands

**Keywords:** Genetic transduction, Genetic vectors, Adult stem cells

## Abstract

The lack of efficient gene transfer methods into primary human pancreatic exocrine cells hampers studies on the plasticity of these cells and their possible role in beta cell regeneration. Therefore, improved gene transfer protocols are needed. Lentiviral vectors are widely used to drive ectopic gene expression in mammalian cells, including primary human islet cells. Here we aimed to optimize gene transfer into primary human exocrine cells using modified lentiviral vectors or transduction conditions. We evaluated different promoters, viral envelopes, medium composition and transduction adjuvants. Transduction efficiency of a reporter vector was evaluated by fluorescence microscopy and flow cytometry. We show that protamine sulfate-assisted transduction of a VSV-G-pseudotyped vector expressing eGFP under the control of a CMV promoter in a serum-free environment resulted in the best transduction efficiency of exocrine cells, reaching up to 90% of GFP-positive cells 5 days after transduction. Our findings will enable further studies on pancreas (patho)physiology that require gene transfer such as gene overexpression, gene knockdown or lineage tracing studies.

## Introduction

Beta cell replacement therapy is a promising treatment for patients with type 1 diabetes mellitus (T1DM)^1^. However, the lack of donor organs prevents the widespread use of this treatment. It is hypothesized that the exocrine compartment of the human pancreas may harbor a progenitor cell population that can develop towards beta cells^2^. Rodent models of diabetes and pancreatic injury, however, demonstrate contradictory results with regards to the exocrine-derived beta cell neogenesis capacity of the adult pancreas^3–8^, and the findings obtained in rodent studies are often not confirmed with human cells^9^. Such discrepancies highlight the need for studies on primary human pancreas cells. Single-cell transcriptomics data of human adult pancreatic cells indicates the duct compartment as a potential source for beta cell progenitor cells^10^. In addition, we previously reported long-term expansion of adult human exocrine tissue generating organoids harboring progenitor cells with endocrine differentiation potential^11^. Yet, the lack of reliable lineage tracing systems hampers studies on the identification of the origin of these newly formed endocrine cells, as endocrine contamination in heterogeneous starting populations cannot be fully excluded. Therefore, the development of efficient gene transfer methods into primary human pancreatic exocrine cells is crucial to allow the assessment of the plasticity of these cells and their possible role in human beta cell regeneration.

Several virus-based gene transfer methods into pancreatic cells are available. Adenoviral vectors have been used previously in primary pancreatic cells^12–17^, but this approach is limited by the non-integrating nature of these viruses, which result in a transient effect of the transduction. Moreover, the large amounts of viral particles necessary for efficient transduction is associated with increased cytotoxicity^13^. Lentiviral vectors have the potential to transduce a wide variety of both dividing and non-dividing cell types with long-term and stable expression of transgenes, including cells that are difficult to transfect such as neuronal and glial cells^18–20^. Also in clinical studies, lentiviral gene modification of autologous hematopoietic stem cells has demonstrated small successes in the treatment of monogenetic blood diseases^21–24^. With regards to pancreatic cells, lentiviral vectors have been successfully used to transduce primary human islet cells, enabling studies focused on human beta cell physiology and plasticity^3,25–31^. However, little is known about lentiviral mediated gene transfer to the primary human pancreatic exocrine cells^32^.

In this study, we optimized the transduction conditions of primary human adult exocrine cells with a HIV-1 (human immune deficiency virus 1) based, replication-deficient, lentiviral reporter vector. This novel protocol can be used for improved transgene expression in primary human exocrine tissue, which will enable further investigations on the putative role of these cells in beta cell regeneration.

## Materials and Methods

### Generation of HIV-1-based SIN lentiviral vectors

Third generation self-inactivating HIV-1 based lentiviral vectors were produced as described previously^20^. In short, three helper plasmids (pMDLg-RRE (gag-pol), pRSV-REV and the envelope plasmid) along with the transgene encoding the lentivector were cotransfected overnight into 293T cells using polyethylenimine. The lentiviral vector was pseudotyped with the surface glycoproteins pCMVAXF, pHCMV-G, pHCMV–LCMV-GP, pRRVpcDNA3.1Zeo+ and pHCMV–RabiesG, coding for the MLV4070Aenv (amphotropic murine leukemia virus), the VSV-G (vesicular stomatitis virus), the LCMV-GP (lymphocytic choriomeningitis virus), the RRV-GP (Ross River virus) and the RV-G (rabies virus) surface glycoproteins, respectively. The LCMV-GP plasmid was kindly provided by dr. von Laer (Innsbruck Medical University, Innsbruck, Austria), the pcDNA3.1Zeo+ plasmid was kindly provided by dr. Sanders (Purdue University, West-Lafayette, United States) and the MLV4070Aenv, RRV-GP, RV-G plasmids were kindly provided by dr. Renner (University of Veterinary Medicine, Vienna, Austria). Medium was refreshed and viral supernatant was harvested after 48 hours and 72 hours and filtered using a 0.45 um filter (Pall Corporation). For concentration, lentiviruses were concentrated by one round of ultracentrifugation at 50.000 g for 120 minutes at 4 °C. After removal of the supernatant, the remaining pellet was resuspended in 1 mL of T50N130E1 buffer (50 mM Tris-Cl, 130 mM NaCl and 1 mM EDTA; pH 7.8) by shaking overnight at 4 °C. Virus was quantified by antigen capture ELISA measuring HIV p24 levels (Zeptometrix) and stored at −80 °C.

### Dissociation and purification of human pancreatic islet-depleted tissue

Pancreatic tissue was obtained from human organ donor pancreata. Islets and exocrine tissue were only studied if they could not be used for clinical purposes and if research consent was present. According to national law, ethics approval is not required for research on donor tissues that cannot be used for clinical transplantation. All methods were carried out in accordance with relevant guidelines and regulations. Mean age of the donors was 51.3 years (range 23–71 years), mean BMI was 25.4 kg/m^2^ (range 21–31 kg/m^2^), and none of the donors had a medical history of diabetes mellitus (Table S1). The donor pancreata were processed at the Good Manufacturing Practice facility of our institute according to the Ricordi method as described previously^33^. The donor pancreata were enzymatically and mechanically digested followed by islet purification using a Ficoll gradient. The islet-depleted tissue contained <5% islets as determined by dithizone staining and was cultured in culture bags containing 4 mL of tissue in 200 mL Dulbecco’s Modified Eagle Medium (DMEM) (Invitrogen) supplemented with 10% heat-inactivated fetal calf serum (FCS) (Bodinco), 100 U/mL penicillin (Invitrogen) and 100 μg/mL streptomycin (Invitrogen). After overnight culture, islet depleted tissue was dissociated with trypsin and filtered using 70 μm filters (Miltenyi Biotech). For duct purification, magnetic-activated cell sort (MACS) was performed on dissociated islet-depleted tissue using the ductal cell surface marker CA19-9 (Carbohydrate antigen 19-9) as described previously^34,35^. Briefly, cells were incubated with an antibody against CA19-9 (1:200; Invitrogen) for 30 mins and afterwards with anti-mouse IgG1 microbeads (1:250; Miltenyi Biotech), before magnetic sorting with LS-columns and a QuadroMACS separator (Miltenyi Biotech).

### Lentiviral transduction of human pancreatic exocrine cells

**Figure 1:**
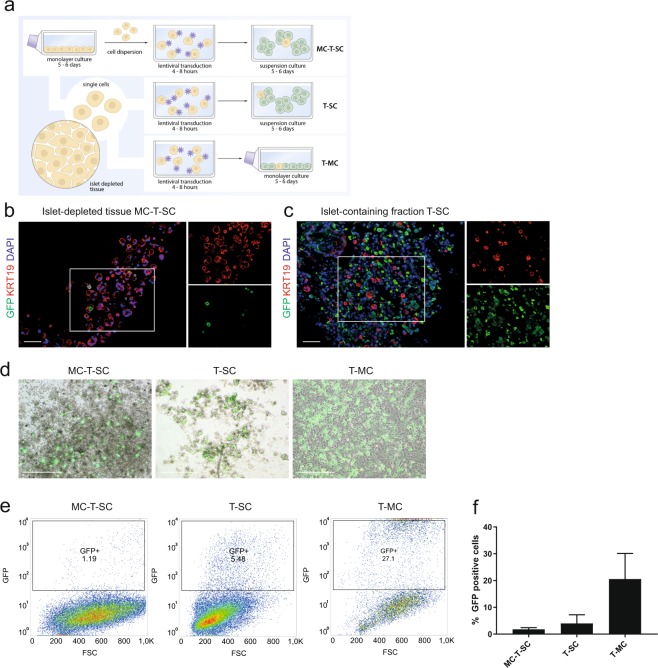
Human islet-depleted lentiviral transduction efficiency can be improved by transducing cells before culture. (**a**) Illustration of the transduction with cultured or freshly isolated human islet depleted tissue. (**b**) Immunofluorescent staining for KRT19 (ductal marker, red) and GFP (green) of human dissociated islet-depleted tissue after monolayer expansion transduced with CMV-GFP in the standard condition prior to suspension culture (‘MC-T-SC’) at day 5 post-transduction, demonstrating sporadic expression of GFP^+^ cells. Scale bar = 100 μm. (**c**) Immunofluorescent staining for KRT19 (ductal marker, red) and GFP (green) of human dissociated islet-containing fractions (30% purity) transduced with CMV-GFP in the standard condition prior to suspension culture (‘T-SC’) at day 5 post-transduction, showing a large proportion of GFP^+^KRT19^-^ cells. Scale bar = 100 μm. (**d**) Brightfield images with GFP overlay of human dissociated islet-depleted tissue transduced with CMV-GFP in the standard condition at different time points during culture at day 5 post-transduction. Scale bar “MC-T-SC“ and “T-SC” = 500 μm. Scale bar “T-MC” = 200 μm. (**e**) Representative flow cytometry plots showing GFP versus forward scatter of human dissociated islet-depleted tissue transduced with CMV-GFP in the standard condition at different time points during culture at day 5 post-transduction. (**f**) Flow cytometry quantification of the GFP-positive fraction of human dissociated islet-depleted tissue transduced with CMV-GFP in the standard condition at different time points during culture at day 5 post-transduction, showing the highest fraction of GFP-positive cells in the condition with cells transduced before monolayer expansion (n = 2–3; mean ± SEM).

After assessment of cell viability with trypan blue, viral supernatant was added to the cells in an ultra-low attachment plate or dish (Costar, Corning) and cells were incubated with viral supernatant for 4 hours, 8 hours or overnight. For the default transduction condition a vesicular stomatitis virus glycoprotein (VSV-G)-pseudotyped cytomegalovirus-green fluorescent protein (CMV-GFP) vector was used at a multiplicity of infection (MOI) of 2, and transduction was performed in serum-rich medium supplemented with polybrene, unless specified otherwise. Serum-free transductions were performed in DMEM supplemented with 0.1% human albumin (Sanquin) and 1x Insulin-Transferrin-Selenium (ITS) (Sigma Aldrich). The polycation agents used were polybrene 8 µg/mL, protamine sulfate 4 µg/mL or DEAE-Dextran 8 µg/mL. The transduction volume can be of influence on the transduction efficiency, therefore the transduction volume was kept uniform throughout all experiments, with a volume of 135.5 µl/cm^2^ of growth area of the well/dish the transduction took place in, and with a minimum of 50% of the transduction volume consisting of fresh medium. Transduction efficiency was evaluated of primary pancreatic exocrine cells that were first expanded for 5–6 days in monolayer culture in treated tissue-culture flasks (Corning) prior to transduction. At confluency the cells in monolayer were detached and incubated with viral supernatant in suspension in ultra-low attachment plates. After incubation cells were spun down by centrifuge, and after removal of the viral supernatant cells were washed with PBS and subsequently cultured in suspension in ultra-low attachment plates (Fig. 1a ‘MC-T-SC’). For the two other conditions, fresh islet-depleted tissue was used after a post-isolation overnight culture in culture bags. Cells were dissociated and incubated with viral supernatant in ultra-low attachment plates in suspension. After incubation cells were spun down by centrifuge, and after removal of the viral supernatant cells were washed with PBS and cultured in suspension in ultra-low attachment plates (Fig. 1a ‘T-SC’), or as monolayer on treated tissue-culture flasks (Fig. 1a ‘T-MC’). Cells in all conditions were cultured in Endothelial Cell Basal Medium-2 (EBM-2) (Lonza) supplemented with 10% heat-inactivated FCS, 100 U/mL penicillin and 100 µg/mL streptomycin for 5–6 days before analysis. Medium in all conditions was refreshed every two days.

### Flow cytometry

Cells in suspension culture were washed and dissociated with trypsin. For monolayer cultures, cells were detached from the plastic with non-enzymatic cell dissociation solution (Sigma). Dissociated cells were washed with PBS and afterward filtered with a 40-μm filter (Miltenyi Biotec). After fixation with formalin and permeabilization with 0.1% saponin, cells were incubated with antibodies against keratin19 (1:100; Dakocytomation) with the secondary antibody anti-mouse Alexa-Fluor 647 (1:500; Molecular Probes). Endogenous GFP was used for analysis of GFP gene expression. Flow cytometry was performed on a FACScalibur (BD Biosciences) or LSR II (BD Biosciences) and flow cytometry data was analysed with FlowJo X (FlowJo).

### Immunostaining and microscopy

Cells were fixed with formalin and centrifuged at high speed in fluid agar. Agar-containing cell pellets were subsequently embedded in paraffin blocks which were cut into 4-μm sections. Primary antibodies against keratin 19 (KRT19, 1:100; Dakocytomation, 1:250; Abcam) and GFP (1:500; Roche and Molecular probes) were used. Secondary antibodies were Alexa-Fluor 488 and Alexa-Fluor 568 anti-mouse or anti-rabbit (1:1000; Molecular Probes), 4′,6-diamidino-2-phenylindole (DAPI) (Vector) was used for nuclear counterstaining. Immunofluorescent images of paraffin slides were acquired with a DM5500 microscope (Leica). The GFP expression of live cells was evaluated with a CK40 microscope (Olympus) using endogenous GFP expression. Images were processed using Zen Lite software (Zeiss).

## Results

### Transduction efficiency of islet-depleted tissue is higher in freshly isolated exocrine cells as compared to monolayer expanded cells

We first tested the standard protocol for lentivirus transduction of mammalian cells that we successfully used on primary human beta cells in previous experiments^30^. Because it is hypothesized that the putative progenitor cell subpopulation involved in beta cell regeneration is located in the ductal compartment of the pancreas, initial experiments were performed on human pancreatic exocrine cultures that were enriched for ductal cells by monolayer culture on tissue-culture treated plastic, to which the pancreatic ductal cells selectively adhere^36^. Because these preliminary experiments indicated that transduction of human exocrine cells during monolayer expansion was very poor (<1% of GFP-positive cells, data not shown), we tested whether transduction efficiency of human pancreatic exocrine cells could be improved by transduction performed on cells in suspension after they had first been expanded as monolayer culture for 5–6 days. Confluent monolayers were detached, and dispersed cells were transduced with a CMV-GFP vector in standard conditions (in suspension, MOI 2, serum-containing medium supplemented with polybrene overnight or for 8 hours). After medium refreshment, cells were transferred to ultra-low attachment plates for suspension culture and spontaneously aggregated (Fig. 1a ‘MC-T-SC’). The transduction efficiency was low. Immunostainings showed only sporadic expression of the GFP reporter gene (Fig. 1b), and only up to 1.2% of cells were GFP positive as quantified by flow cytometry (Fig. 1f ‘MC-T-SC’, n = 3), suggesting a resilience of primary human ductal cells to lentiviral transduction.

Next, we investigated whether the transduction efficiency could be improved if fresh islet-depleted tissue was used, with only a post-isolation overnight recovery culture in culture bags. Dissociated islet-depleted tissue was transduced with a CMV-GFP in standard conditions (in suspension, MOI 2, serum-containing medium supplemented with polybrene overnight or for 8 hours). After transduction, the transduced cells were then either maintained in suspension culture (Fig. 1a ‘T-SC’) or transferred to monolayer culture (Fig. 1a ’T-MC’). Transduction of islet-containing fractions under T-SC condition resulted in a large fraction of GFP positive cells after transduction, but with only a minor fraction of these GFP-positive cells co-expressing KRT19 (Fig. 1c), confirming a resilience of ductal cells to transduction. Similarly, exocrine cells transduced under these conditions showed an increase in the fraction of GFP-positive cells, limited to 5% of GFP-positive cells 5 days post-transduction (Fig. 1d-f ‘T-SC’, n = 2). Transduced cells transferred to monolayer culture after transduction were able to proliferate and showed up to 30% of cells GFP-positive 5 days after transduction (Fig. 1d–f ‘T-MC’, n = 2). Overall, these results show that transduction efficiency  is improved when freshly isolated exocrine cells are transduced in suspension, but the efficiency is still limited.

### CMV is the strongest constitutive promoter for the transduction of primary humane exocrine cells

**Figure 2:**
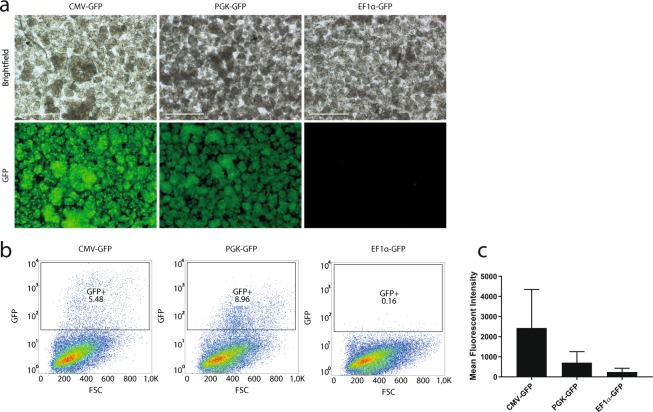
The constitutive CMV promoter yields the brightest GFP positive cells. (**a**) Brightfield and GFP images of human dissociated islet-depleted tissue transduced with CMV-GFP, PGK-GFP or EF1a-GFP in the standard condition prior to suspension culture (‘T-SC’) at day 5 post-transduction, demonstrating brightest GFP expression in cells transduced with CMV-GFP. Scale bar = 200 μm. (**b**) Representative flow cytometry plots showing GFP versus forward scatter of human dissociated islet-depleted tissue transduced with CMV-GFP, PGK-GFP or EF1a-GFP in the standard condition prior to suspension culture (‘T-SC’) at day 5 post-transduction, showing the highest fraction of GFP-positive cells in the culture transduced with PGK-GFP. (**c**) Mean Fluorescent Intensity (MFI) of the GFP-signal of human dissociated islet-depleted tissue transduced with CMV-GFP, PGK-GFP or EF1a-GFP in the standard condition prior to suspension culture (‘T-SC’) at day 5 post-transduction, showing the highest MFI in cells transduced with CMV-GFP (n = 3, mean ± SEM).

We then examined whether the choice of promoter (CMV) could partly explain the low frequency of GFP-positive cells. We tested two other constitutively active promoters of non-viral origin that are often successfully used in lentiviral vectors, the elongation factor 1a (EF1a) and the phosphoglycerate kinase (PGK) promoter. Dissociated islet-depleted tissue was transduced with a promoter-GFP vector in standard conditions (in suspension, MOI 2, serum-containing medium supplemented with polybrene overnight or for 8 hours), and subsequently cultured in suspension (Fig. 1a ‘T-SC’). The GFP intensity was evaluated with fluorescence microscopy (Fig. 2a), showing brightest GFP expression in cells transduced with the CMV-GFP vector. Quantification with flow cytometry indicated a slightly higher frequency of GFP-positive cells upon transduction with the PGK-GFP vector (Fig. 2b), but the intensity of the signal was lower than for CMV-GFP, as illustrated by the difference in mean fluorescence intensity (MFI) (Fig. 2c., n = 3). The EF1a promoter was the weakest promoter, with only sporadic cells expressing GFP. Thus, with CMV being the strongest promoter with brightest GFP-expression and highest MFI in our target cells, this promoter was used for subsequent experiments and optimization of the transduction protocol.

### An increased MOI does not increase the transduction efficiency

To investigate whether a higher MOI could increase transduction efficiency, we compared several MOIs in suspension and monolayer culture. In this experiment, dissociated islet-depleted tissue was first enriched for ductal cells by using MACS for the cell surface marker CA19-9.

After isolation, exocrine cells were transduced with a CMV-GFP lentivirus at a MOI of 2, 7 or 13 in standard condition (in suspension, serum-containing medium supplemented with polybrene overnight or for 8 hours), with subsequent culture in suspension (Fig. 1a ‘T-SC’). Evaluation with microscopy showed that cultures transduced with a higher MOI had fewer aggregates and more single cells (Supplementary Fig. 1a). Also, flow cytometry analysis showed an increase of a side population of cells in the FSC/SSC plot (Supplementary Fig. 1b, n = 1), most likely representing unhealthy cells. In addition, no difference in GFP-expression was observed with fluorescence microscopy or flow cytometry, with approximately 6% of cells GFP-positive 5 days post-transduction with a MOI of 2, 7 or 13.

In the monolayer setup islet-depleted tissue was enriched for CA19-9 cells by MACS. After isolation, cells were plated as monolayer culture and transduced while in monolayer expansion at ~ 40% confluency with a CMV-GFP with a MOI of 2, 4 or 6 in standard conditions (serum-containing medium supplemented with polybrene overnight or for 8 hours). During expansion, these cells showed an increase in cell detachment with increased MOIs, suggesting increased cytotoxicity (Supplementary Fig. 1c, n = 1). Subsequent flow cytometry analysis did not show an increase in the fraction of GFP-positive cells, and moreover indicated a decrease in the KRT19-positive cell fraction of the monolayer cultures transduced with a MOI of 4 or 6 (Supplementary Fig. 1d, n = 1). Because transduction efficiency did not increase with a higher MOI but did seem to have cytotoxic effect, a MOI of 2 was selected as standard MOI for future experiments.

### Increased transduction efficiency with the adjuvant protamine sulfate and serum-free transduction environment

**Figure 3:**
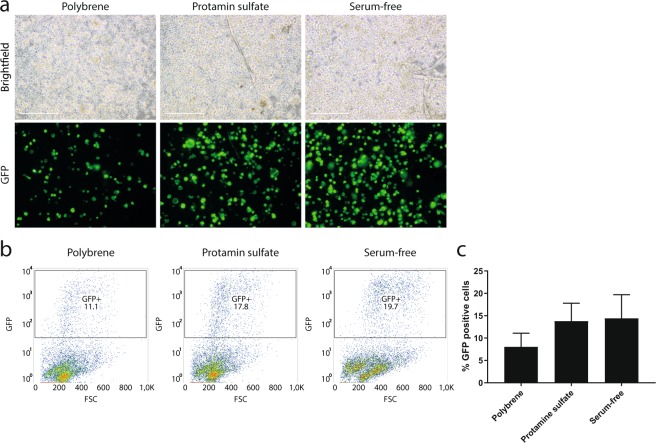
Improved transduction efficiency with the adjuvant protamine sulfate and a serum-free environment without adjuvant. (**a**) Brightfield and GFP images of ductal cells isolated from human dissociated islet-depleted tissue, transduced with CMV-GFP in presence of polybrene, protamine sulfate or in absence of serum and adjuvant prior to suspension culture (‘T-SC’) at day 5 post-transduction. Scale bar = 100 μm. (**b**) Representative flow cytometry plots showing GFP versus forward scatter of ductal cells isolated from human dissociated islet-depleted tissue, transduced with CMV-GFP in presence of polybrene, protamine sulfate or in absence of serum and adjuvant prior to suspension culture (‘T-SC’) at day 5 post-transduction, showing a small increase in the fraction of GFP-positive cells when protamin sulfate or a serum-free condition without adjuvant was used. (**c**) Quantification of GFP-positive cells by flow cytometry of ductal cells isolated from human dissociated islet-depleted tissue, transduced with CMV-GFP in the presence of polybrene, protamine sulfate or in the absence of serum and adjuvant with subsequent suspension culture (‘T-SC’) at day 5 post-transduction, showing an increase to 15% of GFP-positive cells (n = 2, mean ± SEM).

**Figure 4:**
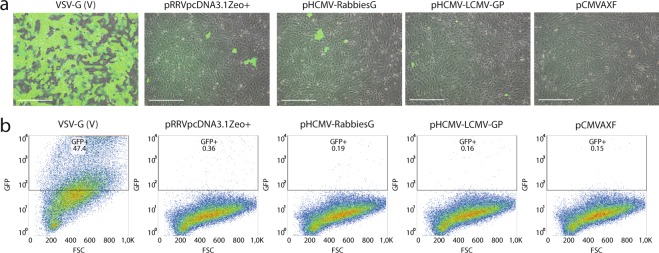
Pseudotyping with the surface glycoproteins of vesicular stomatitis virus yields the highest transduction efficiency. (**a**) Brightfield images with GFP overlay of human dissociated islet-depleted tissue, transduced in serum-free medium in the presence of protamine sulfate with CMV-GFP pseudotyped with the VSV-G, RR-GP, RRV-GP, LCMV-GP or MLV4070Aenv with subsequent monolayer culture (‘T-MC’) at day 5 post-transduction, with the most GFP^+^-cells observed with the VSV-G pseudotyped vector and sporadic GFP^+^-cells with the other pseudotyped vectors. Scale bar = 200 μm. (**b**) Flow cytometry data showing GFP versus forward scatter of human exocrine cells transduced in serum-free medium in the presence of protamine sulfate with CMV-GFP pseudotyped with the VSV-G, RR-GP, RRV-GP, LCMV-GP or MLV4070Aenv with subsequent monolayer culture (‘T-MC’) at day 5 post-transduction. A large fraction of GFP-positive cells was observed when the VSV-G pseudotyped vector was used (n = 3).

Another potential factor that may affect the transduction efficiency of primary cells is the adjuvant used during the transduction and the presence of serum. We first tested the effect of the commonly used adjuvants polybrene and DEAE-dextran on dissociated islet-depleted tissue during standard transduction before suspension culture (Fig. 1a ‘T-SC’). There was no improvement in GFP-expression with polybrene or DEAE-dextran compared to the control without adjuvant (Supplementary Fig. 2a–c, n = 2–3). Next, we tested the effect of protamine sulfate as adjuvant and a serum-free transduction environment on the transduction efficiency. In this experiment, CA19-9 cells were isolated from dissociated islet depleted tissue with MACS. After isolation, the adjuvants polybrene, protamine sulfate and serum-free transduction without adjuvant were compared on exocrine cells transduced with a CMV-GFP in standard conditions (in suspension, MOI 2, serum-containing medium for 4 hours), with subsequent culture in suspension (Fig. 1a ’T-SC’). The adjuvant protamin sulfate in the presence of serum, or a serum-free transduction environment without adjuvant, appeared to increase the percentage of GFP-positive cells to 15% of the ductal cells (Fig. 3a–c, n = 2), and the same effect was also seen on non-purified dissociated islet depleted tissue (data not shown, n = 2). Subsequently, we investigated the effect of these transduction conditions combined on dissociated islet-depleted tissue prior to enrichment for ductal cells by monolayer culture on tissue-culture treated plastic. The combination of protamine sulfate combined with serum-free transduction on non-purified dissociated islet-depleted tissue transduced before monolayer culture (Fig. 1a ‘T-MC’) further increased the number of GFP-positive cells up to 50% (Fig. 4a,b ‘VSV-G (V), n = 4). Therefore, this method was used for subsequent experiments.

### Pseudotyping with the envelope of vesicular stomatitis virus yields the highest transduction efficiency

To investigate whether cell entry of the lentivirus vector in primary exocrine cells could be a limitation for efficient transduction we tested envelopes from other viruses. The envelopes tested were derived from Ross River virus, rabies virus, lymphocytic choriomeningitis virus, amphotropic murine leukemia virus, and were compared to the most commonly used vesicular stomatitis virus envelope. Dissociated islet-depleted tissue was transduced with a pseudotyped CMV-GFP vector in serum-free medium supplemented with protamine sulfate for 4 hours, with subsequent monolayer expansion (Fig. 1a ‘T-MC’). We observed a large difference in the fraction of GFP-positive cells, with 47% of GFP-positive cells when the VSV-G pseudotyped lentiviral vector was used compared to only sporadic GFP-expressing cells when one of the other pseudotyped vector was used (Fig. 4a,b, n = 3). Based on these results, lentiviral vectors for the primary exocrine cells were pseudotyped with the VSV-G envelope for future experiments.

### Ultracentrifugation of viral supernatant further increases the efficiency of transduction

**Figure 5:**
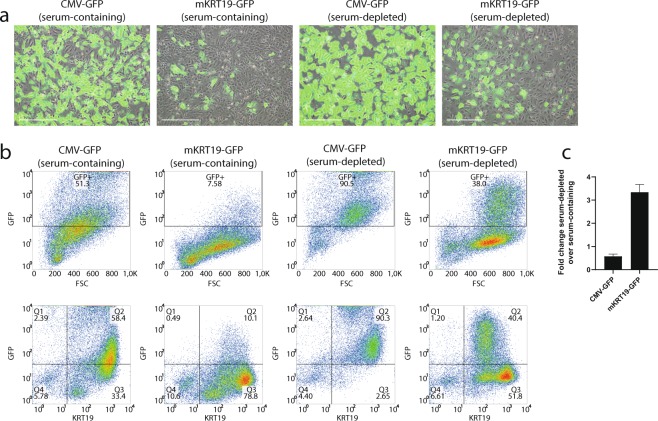
Higher transduction efficiency by serum-depletion of viral supernatant. (**a**) Brightfield and GFP overlay images of human dissociated islet-depleted tissue transduced with CMV-GFP or mKRT19-GFP serum-depleted or serum-containing viral supernatant at a MOI of 2 in serum-free medium in presence of protamin sulfate with subsequent monolayer culture (‘T-MC’) at day 5 post-transduction. Scale bar = 200 μm. (**b**) Flow cytometry plots showing GFP versus forward scatter, and GFP versus KRT19 (ductal marker), of human dissociated islet-depleted tissue transduced with CMV-GFP or mKRT19-GFP serum-depleted or serum-containing viral supernatant at a MOI of 2 in serum-free medium in presence of protamin sulfate with subsequent monolayer culture (‘T-MC’), at 5 days post-transduction showing an increase up to 90% of GFP-positive cells when serum-depleted CMV-GFP viral supernatant was used. (**c**) Fold change of the GFP-positive fraction when human dissociated islet-depleted tissue was transduced with a serum-depleted as compared to serum-containing viral supernatant of a CMV-GFP or a mKRT19-GFP construct at a MOI of 2 in presence of protamine sulfate with subsequent monolayer culture (‘T-MC’) at 5 days post-transduction (n = 3, mean ± SEM).

In order to further deplete the lentiviral transduction conditions of serum and other potentially inhibiting factors that might have been secreted by the lentivirus producing cell line, viral supernatant was concentrated by ultracentrifugation and the supernatant containing medium and serum was removed. Dissociated islet-depleted tissue was transduced with serum-containing virus supernatant or serum-depleted virus supernatant at a MOI of 2 in serum-free medium supplemented with protamine sulfate for 4 hours, prior to monolayer expansion (Fig. 1a ‘T-MC). The use of serum-depleted CMV-GFP viral supernatant increased transduction efficiency 0.6-fold to a maximum of 90% of GFP-positive cells, compared to a maximum of 51% of GFP-positive cells with the CMV-GFP serum-containing viral supernatant (Fig. 5a–c ‘CMV-GFP (serum-containing) and CMV-GFP (serum-depleted)’ n = 3).

Finally, in an attempt to develop a ductal cell specific lineage tracing system, we compared specificity and efficiency of various promoters from genes known to be specifically expressed in ductal cells in the human pancreas: 380 bp mouse KRT19 promoter^37^, 732 bp human KRT19 promoter^38^, 2952 bp human KRT19 promoter^39^, and a human carbonic anhydrase II (hCAII) promoter^4,40^. The 380 bp mouse KRT19 (mKRT19) promoter displayed the most promising results with regards to transduction efficiency and specificity on KRT19-positive and KRT19-negative cell lines (Supplemental Fig. 3 and data not shown). Serum-containing viral supernatant of the mKRT19-GFP lentivirus vector was subsequently compared to the mKRT19-GFP serum-depleted viral supernatant on primary human exocrine tissue. The frequency of GFP-positive cells was lower compared to the strong ubiquitous CMV promoter, but we could increase the transduction efficiency 3.4-fold from a maximum of 8% of GFP-positive cells with the serum-containing viral supernatant, to a maximum of 40% of GFP-positive cells using serum-depleted viral supernatant (Fig. 5a–c, n = 3). Importantly, only 1.2% of GFP-positive cells were KRT19-negative, suggesting a good specificity of the construct in these islet-depleted preparations. Next, we tested the specificity of these construct on islet-containing fractions, which also contain endocrine cells next to the exocrine cells. These additional experiments showed a higher frequency (up to 9%) of GFP-positive cells, that were not ductal cells (KRT19-negative) when mKRT19-GFP and hCAII-GFP constructs were used (Supplemental Fig. 4a–c, n = 3). We therefore considered these promoters to be insufficiently specific for application in lineage tracing studies. Yet, altogether our data indicate that ultracentrifugated viral supernatant combined with serum-free transduction condition and protamin sulfate constitute optimal conditions to efficiently transduce primary human exocrine cells.

## Discussion

An alternative source of beta cells for beta cell replacement therapy in type 1 diabetes mellitus is urgently needed as the availability of human pancreas donor-material is too limited. Pancreatic ductal cells are an attractive alternative source for the generation of new beta cells, but it remains unclear whether human pancreatic ductal cells can act as beta cell progenitor cells due to the lack of an efficient lineage tracing system. In contrast to primary human islet cells, primary human ductal cells are highly resilient to lentiviral transduction by standard protocol. Various factors can strongly influence lentiviral transduction. Here we systematically tested various factors such as the timing of transduction, promoters (both ubiquitous and cell type specific), pseudotyping of the lentivirus vector, MOI and the presence of serum or an adjuvant.

First, we looked at host factors that could be of influence on transduction efficiency. We found that cellular state during culture was a critical point of influence on transduction efficiency, with the highest transduction efficiency obtained by using fresh exocrine cells transduced in suspension. Possible reasons for this difference in transduction efficiency might be a change in cellular receptors after adhesion and expansion of these cells, limiting viral entry or post entry events. Cellular adhesion and timing of transduction are also known to play a role in the cellular events controlling viral entry and/or integration in hematopoietic stem cells, which is reflected in transduction efficiency differences depending on the timing, type and pre-stimulation of the culture^41–45^.

Secondly, we looked at the lentiviral vector characteristics that could be important for efficient transduction. We found that the best lentiviral vector for our target cells included a CMV-promoter to drive the expression of the transgene, combined with a vesicular stomatitis virus glycoprotein for cell entry. Since the strength and expression efficiency of a constitutive promoter is variable and dependent on the type of cell to be transduced, it is necessary to compare different promoters in order to select the strongest and most efficient promoter for the cell of interest, which has been illustrated by several published systematic promoter comparisons^46–49^. In attempt to develop a lineage tracing system we also evaluated several promoters of genes known to be specific for the pancreatic ductal cells (KRT19 and CAII), but specificity of these promoters was not specific enough for a lineage tracing system to investigate duct cell fate during different culture conditions. In addition, the envelope which allows the entry of the virus into the cells could also limit transduction efficiency. Pseudotyping of lentiviral vectors by adding surface glycoproteins of other viruses can alter the tropism of a lentiviral vector and potentially increase the amount of specific cellular targets that can be transduced^50^. Our data indicate that transduction efficiency of our target cells was not improved when lentiviral vectors pseudotyped with different envelopes were used, as compared to the standard VSV-G envelope. Finally, we identified the adjuvant protamin sulfate in combination with serum-free medium and ultracentrifugated lentiviral supernatant as the best environment for the transduction of exocrine cells. The presence of positively-charged polycation agents reduces electrostatic repulsion forces between the negatively charged cell and lentiviral particles, and thus improves transduction efficiency. The effect of these adjuvants is dependent on the type of cell that is transduced, and the lentiviral construct that is used^51,52^. We also observed the variability of this adjuvant effect in our experiments, with no effect on transduction efficiency when the widely used polybrene and DEAE-dextran were evaluated as adjuvant for our target cells. In addition, the presence of serum and other unknown factors that might have been secreted by the lentivirus producing cell lines during the transduction can also affect the efficiency of transduction. Postulated mechanisms include an attenuated effect of positive polycation agents by the negatively charged proteins present in serum and medium, or virus particle aggregation due to proteins, resulting in viral aggregates with less infectious capacity compared to single virus particles^53^. For example, hematopoietic stem cell transduction efficiency is negatively affected when transduction is performed in conditions supplemented with serum^42,54^.

Altogether, our access to primary human tissue from our islet isolation facility enabled us to systematically evaluate various transduction conditions to improve lentiviral transduction of these exocrine cells. Although shortage of donor tissue limited the number of biological replicates we could include for all the different conditions tested, our data provide lentiviral protocol optimisations that enable more efficient gene transfer into primary human pancreatic exocrine cells. These findings will allow future investigations on the (patho)physiology of pancreatic diseases and could stimulate further studies on their possible *ex vivo* gene therapy options, in addition to enabling studies on the plasticity of human pancreatic exocrine cells and their role in beta cell regeneration.

## Supplementary information

Supplementary Information
