# Supplementary material for: Highly efficient ex vivo lentiviral transduction of primary human pancreatic exocrine cells
Source: Sci Rep. 2019 Nov 1;9:15870. doi: 10.1038/s41598-019-51763-z (PMC6825235; doi:10.1038/s41598-019-51763-z)
Supplement: Supplementary file 1 — Supplementary Information [file 41598_2019_51763_MOESM1_ESM.pdf]

Highly efficient ex vivo lentiviral transduction of primary human pancreatic exocrine cells.

Jeetindra R.A. Balak[1], Natascha de Graaf[1], Arnaud Zaldumbide[2], Ton J. Rabelink[1], Rob C. Hoeben[2], Eelco J.P. de Koning[1,3], Françoise Carlotti[1*]

Institution(s):

[1] Department of Internal Medicine, Leiden University Medical Center, Albinusdreef 2, 2333ZA, Leiden, the Netherlands

[2] Department of Cell and Chemical Biology, Leiden University Medical Center, Albinusdreef 2, 2333ZA, Leiden, the Netherlands

[3] Hubrecht Institute, Uppsalalaan 8, 3584CT, Utrecht, the Netherlands

* Corresponding author: F.Carlotti@lumc.nl; Tel.: +31-71-5262011

Supplementary Information includes:

Supplementary Table 1

Supplementary Figure 1

Supplementary Figure 2

Supplementary Figure 3

Supplementary Figure 4

Supplementary Table 1: **Donor characteristics**

Donor number	Sex	Age (years)	BMI (kg/m2)	Cause of death
1	F	61	23	Trauma
2	M	35	24	Stroke
3	F	71	21	Trauma
4	M	31	24	Trauma
5	M	41	22	Trauma
6	M	62	24	Stroke
7	F	55	21	Stroke
8	M	23	22	Trauma
9	M	53	25	Stroke
10	F	52	22	Stroke
11	M	36	25	Meningitis
12	F	57	28	Pulmonary embolism
13	M	72	25	Cardiac arrest
14	M	52	28	Stroke
15	M	39	26	Suicide
16	M	66	31	Stroke
17	F	45	24	Brain tumor
18	F	69	36	Trauma
19	M	47	23	Stroke
20	F	55	29	Stroke
21	M	56	30	Stroke

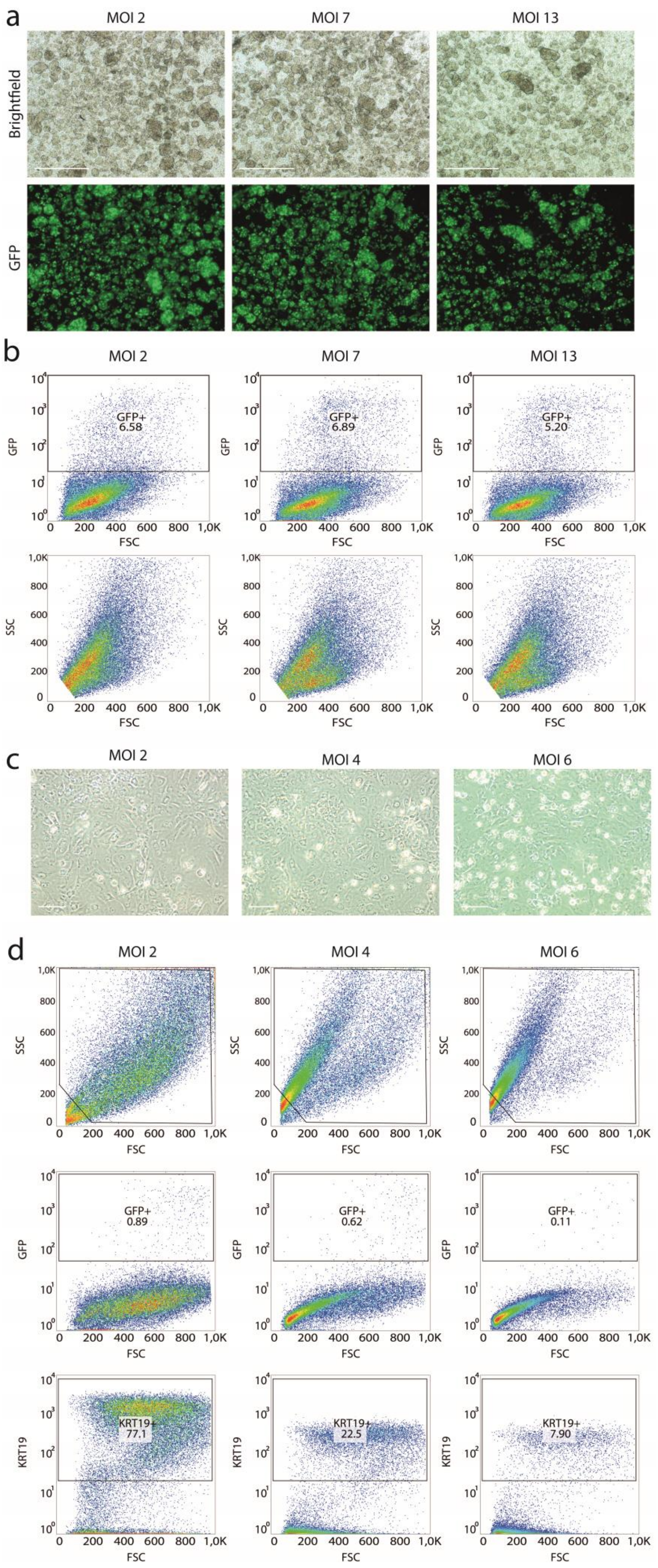

a
MOI 2
MOI 7
MOI 13
Brightfield
GFP
b
MOI 2
MOI 7
MOI 13
GFP+
6.58
GFP+
6.89
GFP+
5.20
GFP
SSC
FSC
c
MOI 2
MOI 4
MOI 6
d
MOI 2
MOI 4
MOI 6
SSC
FSC
GFP+
0.89
GFP+
0.62
GFP+
0.11
GFP
KRT19+
77.1
KRT19+
22.5
KRT19+
7.90
KRT19

Supplementary Figure 1. **An increased MOI does not yield a higher percentage of GFP-positive cells.**

(a) Brightfield and GFP images of ductal cells isolated from dissociated islet-depleted tissue transduced with CMV-GFP in the standard condition with a MOI of 2, 3 or 13, prior to suspension culture ('T-SC') at day 5 post-transduction, demonstrating no difference in GFP expression between the different MOIs and a decreased aggregation at higher MOIs. Scale bar = 200 μm. *(b)* Flow cytometry showing GFP versus forward scatter and side scatter versus forward scatter of ductal cells isolated from dissociated islet-depleted tissue transduced with CMV-GFP in the standard condition with a MOI of 2, 3 or 13, prior to suspension culture ('T-SC') at day 5 post-transduction, confirming the microscopy findings with no increase in the percentage of GFP^{+}-cells and an increase of a side population when a higher MOI was used. (n=1). *(c)* Brightfield images of ductal cells isolated from dissociated islet-depleted tissue and transduced during monolayer expansion with CMV-GFP at a MOI of 2, 4 and 6 in serum-rich medium supplemented with polybrene at day 4 post-transduction, demonstrating increased detachment of ductal cells when a higher MOI is used. Scale bar = 50 μm. *(d)* Flow cytometry showing side scatter versus forward scatter, GFP versus forward scatter and KRT19 (ductal marker) versus forward scatter, of ductal cells isolated from dissociated islet-depleted tissue and transduced during monolayer expansion with CMV-GFP at a MOI of 2, 4 and 6 in serum-rich medium supplemented with polybrene at day 4 post-transduction, showing an increase of a side population of cells, no increase of the GFP-positive fraction and a decrease in the KRT19-positive fraction when higher MOIs were used. (n=1)

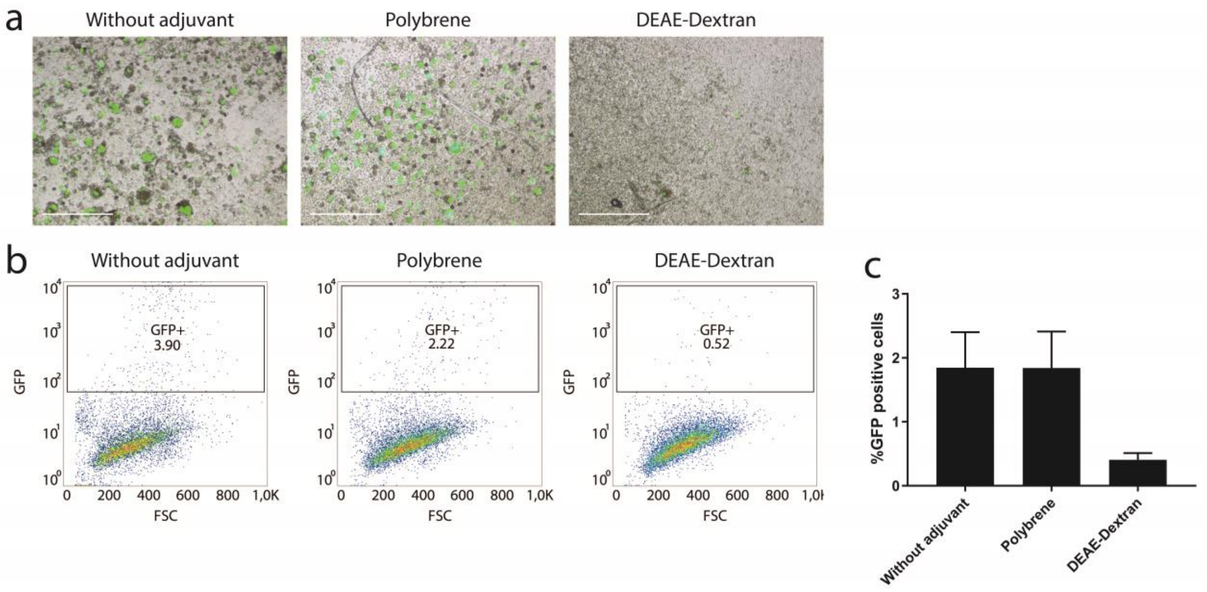

Supplementary Figure 2: **No increase in transduction efficiency with the use of the polycation agents polybrene or DEAE-Dextran.**

(a) Brightfield and GFP images of dissociated islet-depleted tissue transduced with CMV-GFP in the standard condition without adjuvant, or in presence of polybrene or protamine sulfate, prior to suspension culture ('T-SC') at day 5 post-transduction, showing no increase in GFP^+-cells with the adjuvants. Scale bar = 500 μm. (*b*) Representative flow cytometry plots showing GFP versus forward scatter of dissociated islet-depleted tissue transduced with CMV-GFP in the standard condition without adjuvant, or in presence of polybrene or protamine sulfate, prior to suspension culture ('T-SC') at day 5 post-transduction. *(c)* Quantification of the GFP-fraction of dissociated islet-depleted tissue transduced with CMV-GFP in the standard condition without adjuvant, or in presence of polybrene or protamine sulfate, prior to suspension culture ('T-SC') at day 5 post-transduction (n=2-3, mean ± SEM).

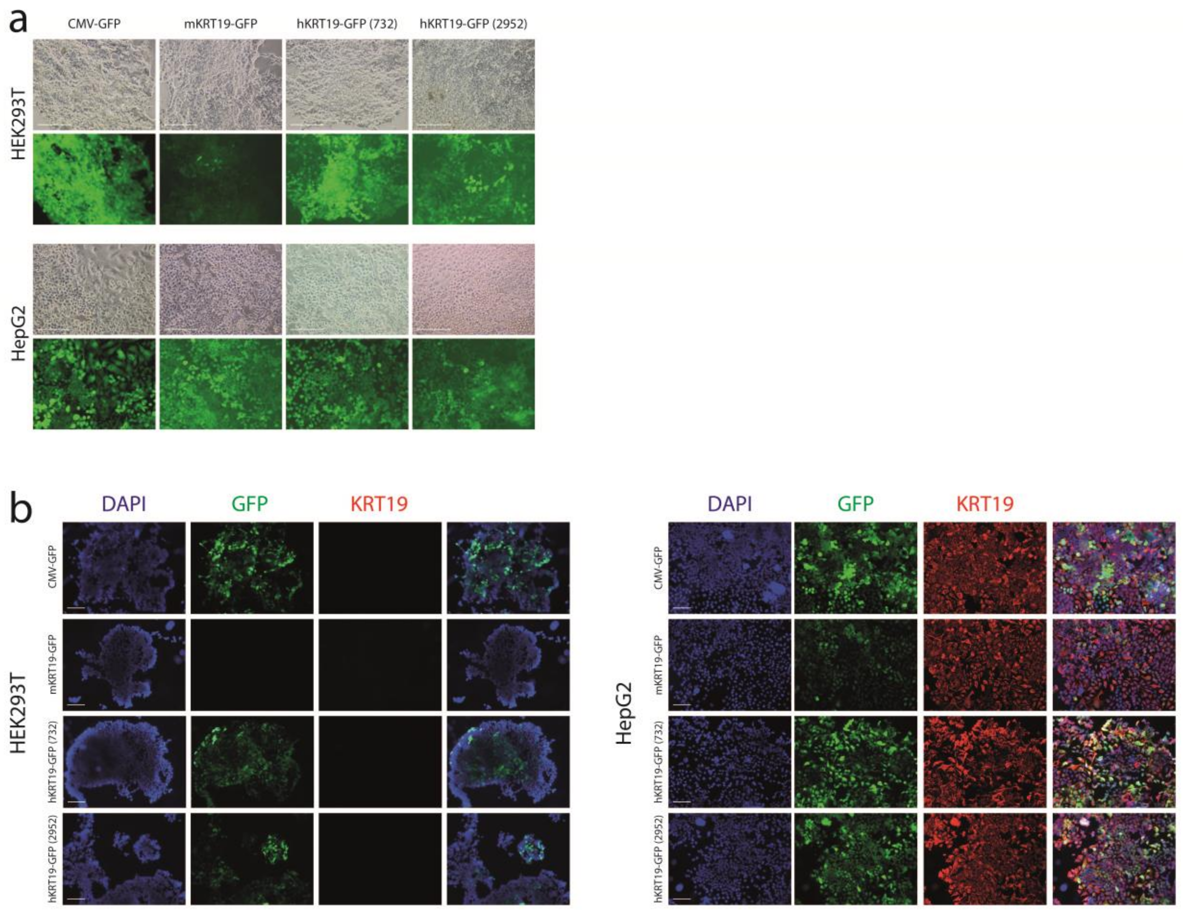

Supplementary Figure 3: **The mKRT19-GFP construct is the most specific construct when tested on KRT19 negative and KRT19 positive cell lines.**

(a) Brightfield and GFP images of the HEK293T cell line (KRT19 negative) and the HepG2 cell line (KRT19 positive) transduced in suspension with a CMV-GFP, mKRT19-GFP, hKRT19 (720bp)-GFP or hKRT19 (2952bp)-GFP construct at a MOI of 2 in serum-containing medium without adjuvants before culture in monolayer on glass coverslip ('T-MC') at day 4 post-transduction. The least amount of GFP expression is observed in the HEK293T cell line when the mKRT19-GFP construct was used, suggesting good specificity of this construct for KRT19 expressing cells. Scale bar = 200 μm. (n=3) *(b)* Immununofluorescent staining of HEK293T cell line (KRT19 negative) and the HepG2 cell line (KRT19 positive) transduced in suspension with a CMV-GFP, mKRT19-GFP, hKRT19 (720bp)-GFP or hKRT19 (2952bp)-GFP construct at a MOI of 2 in serum-containing medium without adjuvants stained for KRT19 (red, ductal marker), and GFP (green), demonstrating the least GFP expression in the HEK293T cell lines when transduced with the mKRT19-GFP, confirming the findings observed with brightfield microscopy. Scale bar = 200 μm. (n=2).

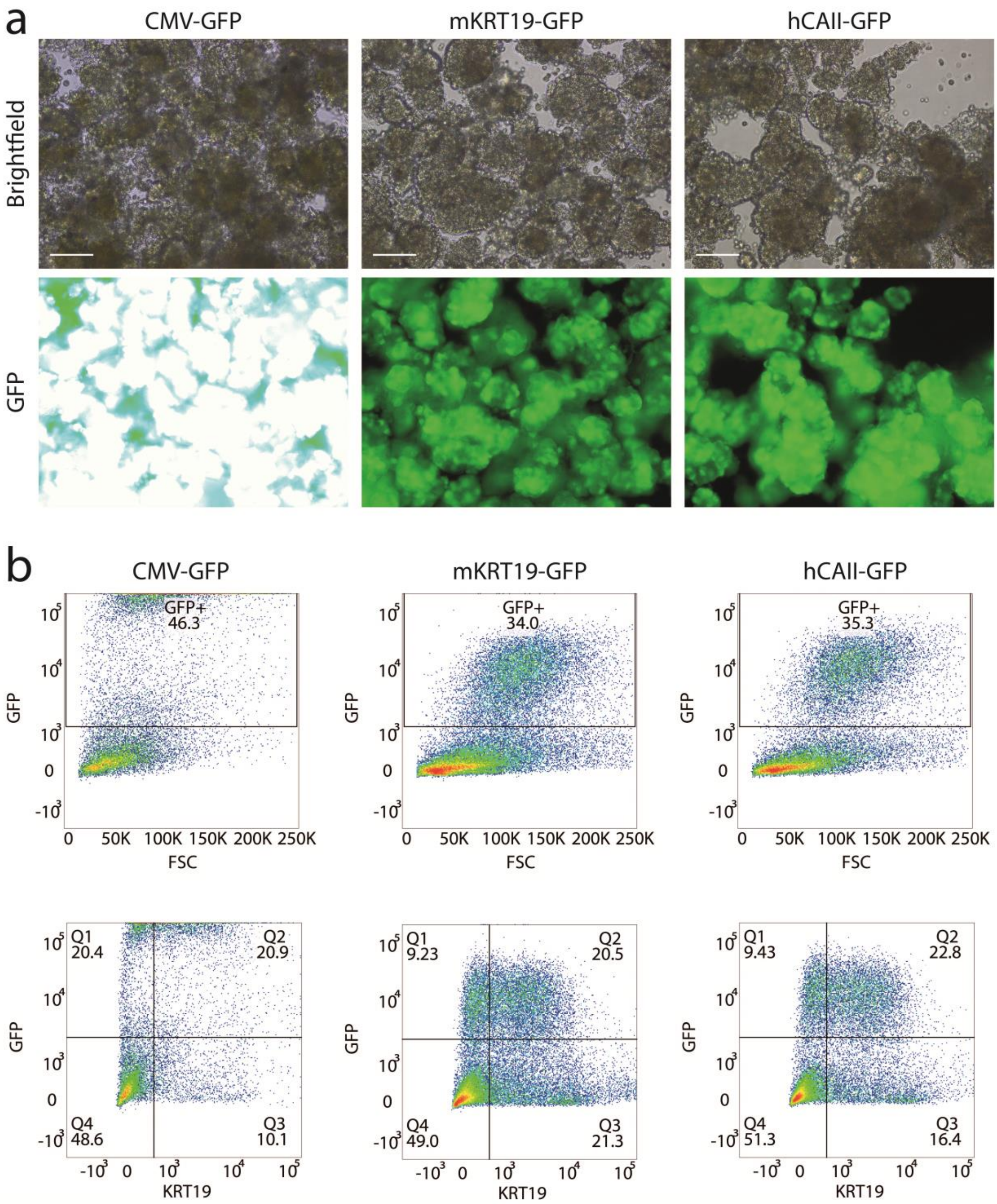

Supplementary Figure 4: **The mKRT19-GFP and hCAII-GFP are not specific for ductal cells in islet-containing fractions.**

(a) Brightfield and GFP images of human dissociated islet-containing fractions (50% purity) transduced in suspension with CMV-GFP, mKRT19-GFP or hCAII-GFP construct at a MOI of 2 in serum-free medium supplemented with protamin sulfate, prior to culture in suspension ('T-SC') at day 5 post-transduction. Scale bar = 50 μm. (*b*) Representative flow cytometry plots showing GFP versus forward

scatter and GFP versus KRT19 (ductal marker) of human dissociated islet-containing fractions (50% purity) transduced in suspension with CMV-GFP, mKRT19 or hCAII-GFP at a MOI of 2 in serum-free medium supplemented with protamin sulfate, prior to culture in suspension (‘T-SC’) at day 5 post-transduction, demonstrating insufficient specificity for primary human ductal cells with >9% of cells $GFP^{+}/KRT19^{-}$ with the mKRT19-GFP or hCAII-GFP vector (n=3).
